# Telomere length dynamics measured by flow-FISH in patients with obesity undergoing bariatric surgery

**DOI:** 10.1038/s41598-022-27196-6

**Published:** 2023-01-06

**Authors:** Benjamin Rolles, Monica S. V. Ferreira, Margherita Vieri, Karl P. Rheinwalt, Sophia M. Schmitz, Patrick H. Alizai, Ulf Neumann, Tim H. Brümmendorf, Fabian Beier, Tom F. Ulmer, Mareike Tometten

**Affiliations:** 1 Department of Hematology, Oncology, Hemostaseology and Stem Cell Transplantation, Medical Faculty, RWTH Aachen University, Aachen, Germany; 2 Center for Integrated Oncology Aachen Bonn Cologne Duesseldorf (CIO ABCD), Aachen, Germany; 3 Department of Bariatric, Metabolic and Plastic Surgery, St. Franziskus Hospital, Cologne, Germany; 4 Department of General, Visceral and Transplantation Surgery, Medical Faculty, RWTH Aachen University, Aachen, Germany

**Keywords:** Obesity, Biomarkers, Inflammation

## Abstract

Obesity has negative effects on comorbidities, health-related quality of life and survival. Telomere length (TL) changes after bariatric surgery have been reported, but the studies are contradictory, and analyses using state-of-the art techniques for TL measurement, such as flow-FISH, are sparse. We measured TL dynamics via flow-FISH in patients undergoing bariatric surgery and compared their TL with 105 healthy individuals. Patients with obesity who underwent bariatric surgery were included. Lymphocyte and granulocyte absolute and age-adjusted (aa) TL were analyzed by flow-FISH before (preoperative cohort, n = 45) and after surgery (follow-up cohort, n = 35) at month 5.5 ± 3.9 (mean ± standard deviation [SD]). The initial lymphocyte aaTL was significantly shorter (-0.37 kb ± 0.18 kb, *P* = 0.045) in patients with obesity, while the granulocyte aaTL was not different from that in the healthy comparison population (0.28 kb ± 0.17 kb, *P* = 0.11). The telomere dynamics after surgery showed an increase in mean TL in both lymphocytes and granulocytes of patients with a pronounced BMI loss of ≥ 10 kg/m^2^. We did not find any association between TL increase after surgery and age, sex or the type of procedure selected for bariatric surgery. We confirmed that patients suffering from obesity have significantly shorter lymphocyte TL using flow-FISH. Along with and dependent on the degree of weight reduction after bariatric surgery, TL significantly increased in both lymphocytes and granulocytes after a mean of 5.5 months. Our results show that bariatric surgery affects not only body weight but also biomarkers of aging, such as TL.

## Introduction

Telomeres are the end structures of chromosomes and they have a variety of functions, including protection and stabilization of the chromosomal architecture^1^ as well as preserving the integrity and organization of the DNA^1,2^. In vertebrates, telomeres consist of the repeating hexanucleotides 5′-TTAGGG-3′ that extend the end of chromosomes for several kilobases (kb). During cell division, telomeres shorten consecutively^2^ and thereby mirror the degree of replicative aging the individual somatic tissue has undergone^3^. Critically short telomeres have been associated with cellular senescence^4^, the appearance of a senescence-associated secretory phenotype^5^, increased genetic instability^6^ and eventually apoptosis^7^.

Prematurely shortened telomeres can be found in hereditary primary telomere biology disorders (TBDs), such as dyskeratosis congenita (DKC)^8^, as well as in various acquired hematological diseases, such as chronic myeloid leukemia (CML), acute myeloid leukemia (AML) and aplastic anemia (AA)^2,9–13^ due to a substantially increased replicative demand in the disease-affected cellular compartments. Furthermore, many diseases linked to inflammation, cancer and autoimmunity, such as AA and psoriasis, have been reported to be associated with shorter telomeres^13–15^.

In many countries, the number of people with obesity has been increasing for decades^16^, which is associated with the risk of comorbidities such as coronary heart disease, high blood pressure, stroke, diabetes type II, the risk of a severe course of COVID-19 disease and negative impacts on the quality of life^17–19^. Obesity is defined by a body mass index (BMI) of 30 kg/m^2^ or higher^20^ and is categorized into class I (BMI 30.0–34.9 kg/m^2^), class II (BMI 35.0–39.9 kg/m^2^) and class III (BMI ≥ 40.0 kg/m^2^)^21^. Bariatric surgery is indicated for obesity class III and class II with secondary diseases^22^. According to the therapy guidelines on obesity, initial preoperative management consists of interventions regarding nutrition, exercise and behavior^23^. In the absence of a response to conservative therapy, surgical intervention may be the only currently available effective therapy for patients with severe obesity^24^.

Obesity leads to a reduced life expectancy, as confirmed by large meta-analyses^25^. One underlying mechanism for the fatal consequences of severe overweight is oxidative stress and chronic inflammation in adipose tissue^26^. Both mechanisms are known to contribute to premature shortening of telomeres, which in turn leads to a preaged cellular status^27^. Concordantly, patients with obesity have an increased risk of shortened TL, and BMI is negatively associated with TL^28^. However, despite growing evidence that telomere dynamics are affected by obesity, there are conflicting data regarding the state of TL in patients with obesity and the TL dynamics of those who undergo bariatric surgery^27–41^. In this study, we aimed to elucidate the link between TL and obesity by using the gold standard for the measurement of TL, flow-FISH, and to determine whether bariatric surgery has an impact on telomeres as an established biomarker of aging^42^.

## Materials and methods

### Patient cohort

**Figure 1:**
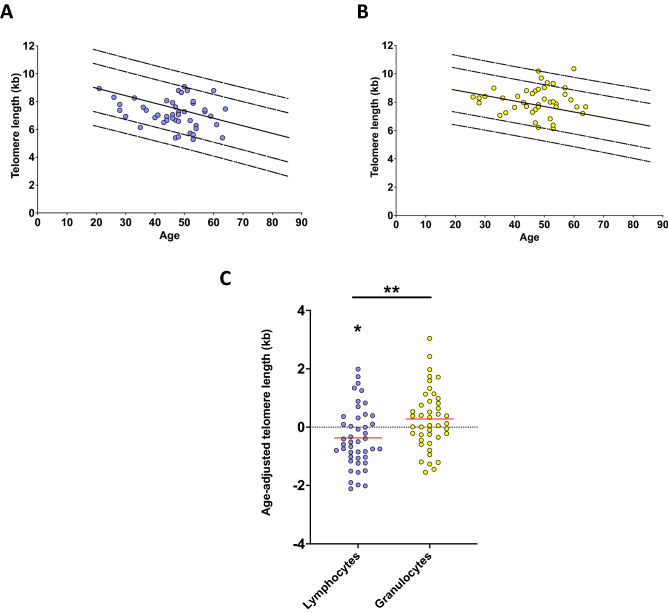
Absolute and age-adjusted (aa) telomere length (TL) in peripheral blood before bariatric surgery. Absolute TL in (**A**) lymphocytes (blue dots; n = 45) and (**B**) granulocytes (yellow dots; n = 43) in patients with obesity before bariatric surgery given in kilobases (kb). The TL of healthy subjects (n = 105) was used to show the 1%, 10%, 50%, 90% and 99% percentile of the TL, respectively (black lines). (**C**) Lymphocytes (blue dots; n = 45) show a mean aaTL of -0.37 kb (*P* = 0.0446) compared to the healthy control population. Granulocytes (yellow dots; n = 43) show a mean aaTL of 0.28 kb (*P* = 0.1108) compard to the healthy control population. Data are displayed as mean (red bar). *Significance of *P* < 0.05, **P < 0.01 (Student’s t-test).

The cohort consisted of 45 patients with obesity undergoing bariatric surgery at the Department of Surgery and Transplantation (University Hospital RWTH Aachen). The inclusion criteria, according to the recommended guidelines for surgical interventions^43^, were BMI ≥ 40 kg/m^2^ or BMI ≥ 35 kg/m^2^ with secondary diseases, minimum age of 18 years and insufficient response during a structured conservative program for weight reduction. Following approval by the ethics committee of the Medical Faculty of RWTH Aachen (EK 206/09) and individual written informed consent, demographic and clinical data were collected. Baseline TL in lymphocytes and granulocytes was measured 11 ± 3.3 days (mean ± SEM) before surgery. In two patients, no granulocyte TL could be measured due to insufficient sample quality. In 35 patients, a follow-up TL measurement was performed 5.5 ± 3.9 months (mean ± SD) after surgery, depending on the individual plans for clinical follow-up visits (Fig. 1). For one patient, no granulocyte TL could be measured after surgery due to insufficient sample quality (Supplementary Fig. 1).

### TL measurement via flow-FISH

At the respective time points, mononuclear cells were isolated from whole blood and frozen at − 80 °C until further processing. TL analysis was performed by flow-FISH as previously described^9–13,44^.

In summary, we prepared the samples after thawing for cell denaturation and stained them with a telomere-specific (CCCTAA)3-peptide nucleic acid (PNA) FITC-labeled FISH probe (Panagene) for DNA hybridization, combined with DNA counterstaining using LDS 751 (Sigma). Fluorescence intensity was measured via an FC-500 (Becton Dickinson) using forward scatter (cell volume) and LDS 751 for identification of the cell subsets (thymocytes, lymphocytes and granulocytes). Autofluorescence values of the respective unstained lymphocytic, granulocytic and thymocytic subpopulations were subtracted from the stained samples, and the mean TL was calculated relative to control cells with a known TL (bovine thymocytes). The whole analysis was carried out in a single-blinded manner in triplicate. Samples from 105 healthy subjects were used as a comparison population for age-adjustment of the TL (aaTL) using a linear regression model. TL is given in kilobases (kb)^2,10–12,44^.

### Statistical analysis

Statistical analysis was performed with Student´s t test. Age-adjusted (aa) TL measurements of the lymphocytes and granulocytes showed a normal distribution with different statistical tests (Shapiro‒Wilk, Kolmogorov‒Smirnov, D’Agostino & Pearson and Anderson‒Darling tests). The age adaptation for TL was carried out using a healthy control cohort of 105 subjects as described previously^45^. Preoperative samples were analyzed with unpaired Student’s t tests to compare the healthy control group with the patient cohort. A paired Student’s t test was used for the analysis of follow-up measurements to reduce interindividual differences. For each patient, a delta TL was calculated and compared pre- and post-surgery. We considered significance at a *P* value * < 0.05, ** < 0.01, and **** < 0.0001. The results are expressed as the mean ± standard deviation (SD). Graphics were created using GraphPad Prism Version 9.1.0, La Jolla, CA, USA.

### Informed consent statement

Informed consent was obtained from all subjects involved in the study.

### Institutional review board statement

The study was conducted according to the guidelines of the Declaration of Helsinki and approved by the Ethics Committee of the Medical Faculty of RWTH Aachen (EK 206/09).

## Results

### Patient characteristics

The mean age of the patient cohort was 46.5 ± 1.5 years (Table 1); 69% were female (n = 31), and 31% were male (n = 14). Different bariatric operations and endoscopic methods were used: sleeve gastrectomy (n = 18, 40%), gastric bypass (n = 24, 53%) and other procedures (intragastric balloon n = 2, 4%; one anastomosis gastric bypass n = 1, 2%, Table 1).

**Table 1:** Characteristics of the patient cohort with obesity.

Category	
Age (y), mean + /- SEM	46.5 (+/−1.5)
**Gender, n (%)**	
Female	31 (69%)
Male	14 (31%)
**Procedure, n (%)**	
Sleeve gastrectomy	18 (40%)
Gastric bypass	24 (53%)
Others*	3 (6%)

The age and surgical or endoscopic procedure of all patients is shown (n = 45).

*Y* years, *SEM* standard error of the mean, *n* number.

*Intragastric balloon (n = 2) and one anastomosis gastric bypass (n = 1).

### Initial TL in patients with obesity was significantly shortened compared to healthy controls

**Figure 2:**
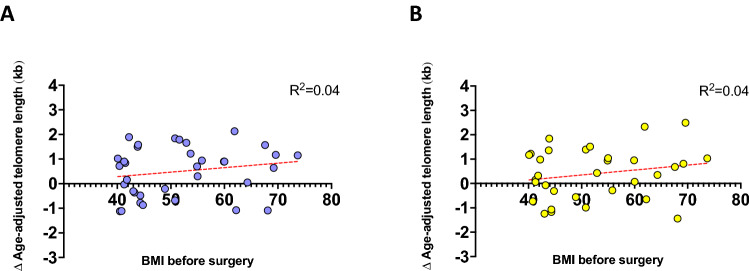
Difference (∆) of the age-adjusted (aa) telomere length (TL) before bariatric surgery. Depicted is the ΔaaTL correlation with the patients’ body mass index (BMI) before surgery. Positive values of the ΔaaTL represent an increase of the TL after surgery in lymphocytes (**A**) (blue dots; n = 35) and granulocytes (**B**) (yellow dots; n = 32). The regression line for lymphocytes (**A**) (*P* = 0.28) and granulocytes (**B**) (*P* = 0.26) shows no significance.

The initial absolute lymphocyte and granulocyte TL (n = 45 and n = 43, respectively) are shown in Fig. 1A (lymphocytes; blue dots) and Fig. 1B (granulocytes; yellow dots), compared to healthy individuals shown as percentile curves as used in previous publications^42,44^. No patient showed TL below the 1st percentile as a potential indicator of an inherited TBD. Compared to healthy controls, the lymphocyte aaTL of all patients before surgery was significantly shortened (-0.37 kb ± 0.18 kb, *P* = 0.045; Fig. 1C), while the granulocyte aaTL was not different (0.28 kb ± 0.17 kb, *P* = 0.11; Fig. 1C). Moreover, the lymphocyte TL was significantly shorter than that of the granulocytes (mean difference 0.65 kb ± 0.23 kb, *P* = 0.0041, Fig. 1C). Healthy individuals also had shorter TLs in lymphocytes than in granulocytes (data not shown). No correlation between preoperative BMI and the difference (Δ) in aaTL in lymphocytes (R^2^ = 0.04, *P* = 0.28) or granulocytes (R^2^ = 0.04, *P* = 0.26) could be detected (Fig. 2).

### TL increase in patients with obesity after bariatric surgery

**Figure 3:**
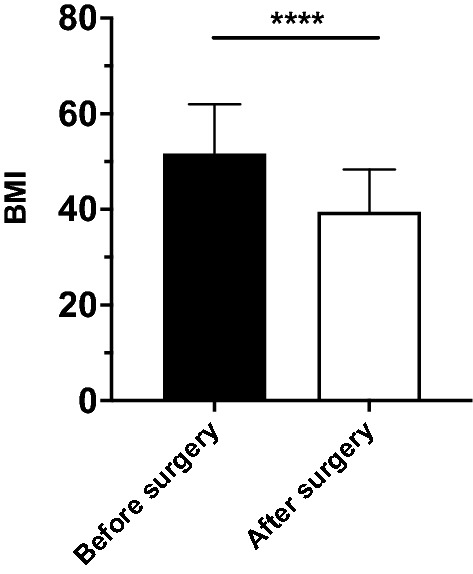
Body mass index (BMI) reduction of the follow-up cohort. BMI before and after surgery of 35 patients is shown. BMI was significantly reduced from 51.7 ± 1.8 kg/m^2^ to 39.5 ± 1.5 kg/m^2^ (*P* < 0.0001; Student’s t-test).

**Figure 4:**
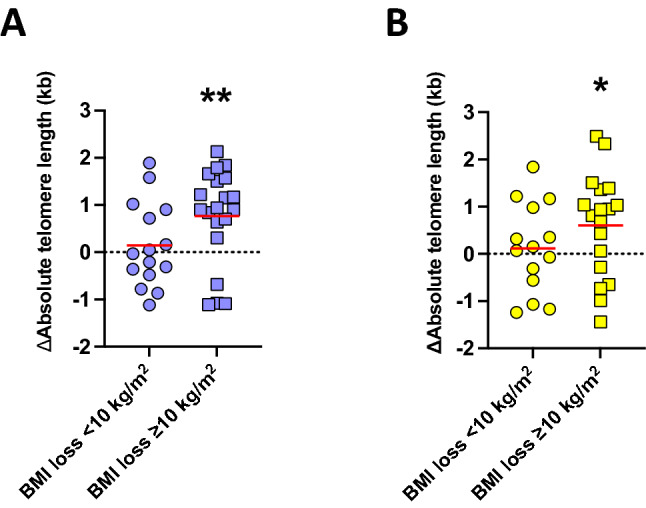
Difference (∆) of the absolute telomere length (TL) of the follow-up population after bariatric surgery (n = 35): (**A**) Lymphocytes (n = 35) and (**B**) granulocytes (n = 32) separated in patients with a body mass index (BMI) loss ≥ 10 kg/m^2^ (shown in squares) or < 10 kg/m^2^ (shown in circles). Patients with BMI loss ≥ 10 kg/m^2^ had a significant increase of TL in lymphocytes and granulocytes. Data are displayed as mean (red bar). *Significance of *P* < 0.05, **P < 0.01 (Student’s t-test).

As expected, BMI was found to be significantly reduced from 51.7 ± 1.8 kg/m^2^ to 39.5 ± 1.5 kg/m^2^ after bariatric surgery (n = 35, *P* < 0.0001; Fig. 3). To estimate the effect of weight loss on telomere dynamics, we defined an absolute BMI loss of ≥ 10 kg/m^2^, representing a change of up to two clinical obesity classes, as a clinically relevant cutoff for weight loss. Moreover, in accordance with the guidelines for the management of overweight and obesity in adults, in which weight loss of at least 20% of the total body weight through bariatric surgery is considered effective^46^, the BMI cutoff value of 10 kg/m^2^ corresponds to a 20% reduction of mean preoperative BMI in our cohort. We observed a significant increase in the absolute lymphocyte TL in patients with a BMI loss ≥ 10 kg/m^2^ (n = 20; *P* = 0.003; Fig. 4A) compared to patients with a BMI loss < 10 kg/m^2^ (n = 15; *P* = 0.54; Fig. 4A). Of note, similar findings were observed for the granulocyte TL. Here, we observed a significant increase in the absolute granulocyte TL in patients with a BMI loss ≥ 10 kg/m^2^ (n = 18; *P* = 0.031; Fig. 4B) compared to patients with a less pronounced weight loss (n = 14; *P* = 0.64; Fig. 4B). Patients with a BMI loss ≥ 10 kg/m^2^ had a mean TL increase of 0.77 kb ± 0.23 kb in lymphocytes and 0.61 kb ± 0.26 kb in granulocytes, corresponding to an improvement in TL calculated as 17.9 life years of TL attrition. In comparison, in patients with a BMI loss < 10 kg/m^2^, no significant changes in lymphocyte TL or granulocyte TL were observed (0.14 kb ± 0.23 kb and 0.12 kb ± 0.25 kb, respectively).

## Discussion

Obesity is associated with chronic inflammation and cardiovascular diseases^17,47–49^, both of which are associated with shortened telomeres and reduced survival^27,28,50^. In line with this, recent studies have linked obesity with shortened telomeres^28,42,51^. In addition, a recent study also showed that pregnancy-related changes in weight gain during pregnancy had an impact on TL^52^. However, data on the association between TL and obesity, particularly telomere dynamics after bariatric surgery, are contradictory. Some studies found shortened telomeres in patients with obesity^28,29^, while others could not confirm these results^30,31^. Similar results on telomere lengthening after successful weight reduction were found, reviewed by Peña et al.^37^. Some data indicate a lengthening of telomeres after weight loss, while some do not^19,32–34,42^. Different timings of TL measurement and different periods of follow-up, ranging from a few months to several years, could explain these contradictory results. However, a major influencing factor is the technique used for TL measurement.

In the vast majority of the studies, PCR techniques were used^29,34,36,39^, and these have several disadvantages, such as reduced accuracy and noncell-specific TL measurement compared to telomere measurement using flow-FISH^45^. In addition, there is no way to differentiate among leukocyte subpopulations when using PCR. This is a significant limitation since lymphocytes (including their naïve and memory subpopulations)^53^ and granulocytes show different absolute values and kinetics of TL during normal physiological aging^2^. Various publications have demonstrated that PCR techniques are limited in the diagnostic potential of telomere biology-associated disorders, particularly when the phenotype is restricted to individual hematopoietic lineages, such as in leukemias^8,45^. Based on individual and prospective evaluations of telomere-associated disease states (e.g., linked to increased replicative aging) as well as in TBD, flow-FISH represents the gold standard of telomere measurement: leukocyte subpopulations such as lymphocytes and granulocytes (as well as their distributions) can be analyzed separately and with high accuracy^45^.

To our knowledge, only one previous study used flow-FISH as a standardized method for TL assessment in the context of bariatric surgery^31^, and our study is the first to specifically investigate the granulocyte compartment. We found a correlation between obesity and TL shortening in lymphocytes, which was not detectable in granulocytes. Following bariatric surgery, the weight reduction was correlated with a significant increase in both lymphocyte and granulocyte TL in those patients who had a BMI change of ≥ 10 kg/m^2^. This cutoff represents a relevant weight reduction, corresponding to at least a 20% weight reduction in baseline body weight^46^.

In the context of obesity, chronic inflammation is probably one of the main mechanisms leading to the consecutive shortening of TLs in immune cells. We hypothesize that the myeloid compartment is also affected by the systemic effects of obesity but to a lesser extent than the lymphatic compartment. The influence of obesity-induced inflammation on the hematopoietic stem cell niche^48^, as well as on myeloid immune cells^54^, has been described previously. Here, we confirmed that weight reduction had beneficial effects on TL in both lymphocytes and granulocytes of obese individuals. As previously shown, adipose tissue, as an important organ of the immune system, is instrumental in impairing immune cells and maintaining chronic inflammation in patients with obesity^49,55^. Lymphocytes in particular play an important role in chronic inflammation and insulin resistance in this patient population^56^. Because of this, we assumed that TL attrition caused by obesity-associated (chronic) inflammation mainly occurs in the lymphocyte compartment. This is also emphasized by the fact that we observed significant differences in lymphocyte and granulocyte TL (Fig. 1C), although this is a phenomenon that can also be observed in healthy individuals. Furthermore, we could not detect any significant change in the TL of the granulocytes in the preoperative cohort, possibly due to the small sample size.

Since we observed a postoperative increase in TL, particularly in patients with significant weight loss, we mechanistically assumed that a significant reduction in adipose tissue is required to reduce systemic inflammation before seeing beneficial effects on telomere biology. We hypothesize that proinflammatory cytokines, such as IL-1, IL-6, and IL-12, which are increased in patients with obesity^57–59^, lead to telomere shortening at the stem cell and progenitor levels. Moreover, an increased activation and proliferation of immune cells, indicated by more white blood cells, especially granulocytes and lymphocytes, in patients with obesity^60^, could be a mechanism of TL shortening. Another explanation for the decrease in TL could be telomere attrition by reactive oxygen species (ROS)^61^ and impairment of telomerase activity in immune cells and their progenitors^62^.

Our study has several limitations that need to be addressed. In addition to a small sample size and being a single-site study, we had a short follow-up time. One could speculate that the positive metabolic changes brought about by weight loss will not be completed in a short time. Further investigation of lymphocyte subpopulations, such as CD4^+^ cells, CD8^+^ cells and T_regs,_ as well as myeloid subpopulations, could reveal the impairment of the immune system more fully.

In conclusion, we showed that patients with obesity have significantly shortened telomeres in only lymphocytes. After bariatric surgery, we observed an increase in both lymphocyte and granulocyte TL in patients with significant weight reduction, arguing for a systemic effect of bariatric surgery and not only a shift between subcompartments. This TL increase following surgery was dependent on the overall degree of BMI reduction. Therefore, we assume that patients with a significant postoperative TL increase will benefit from bariatric surgery to the greatest extent. However, larger prospective studies with longer follow-up periods are needed to validate this assumption.

## Supplementary Information

Supplementary Information.

## Data Availability

The datasets generated during and/or analyzed during the current study are available from the corresponding authors on reasonable request.
