# Supplementary material for: Telomere length dynamics measured by flow-FISH in patients with obesity undergoing bariatric surgery
Source: Sci Rep. 2023 Jan 6;13:304. doi: 10.1038/s41598-022-27196-6 (PMC9818052; doi:10.1038/s41598-022-27196-6)
Supplement: Supplementary file 1 — Supplementary Information. [file 41598_2022_27196_MOESM1_ESM.pptx]

## Slide 1
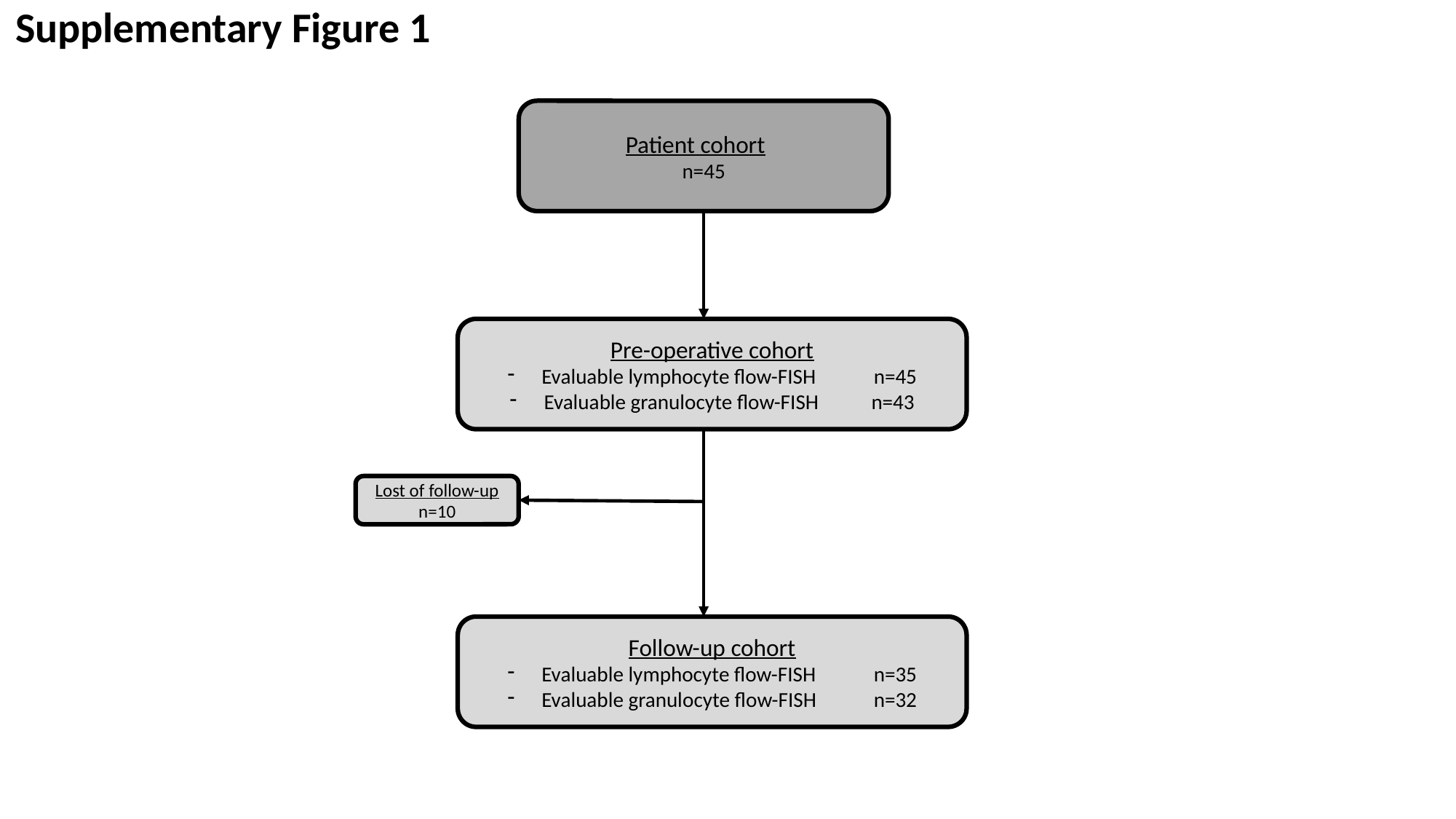

# Supplementary Figure 1
Patient cohort
n=45
Pre-operative cohort
Evaluable lymphocyte flow-FISH	 n=45
Evaluable granulocyte flow-FISH 	n=43
Lost of follow-up
n=10
Follow-up cohort
Evaluable lymphocyte flow-FISH	 n=35
Evaluable granulocyte flow-FISH 	 n=32

## Slide 2
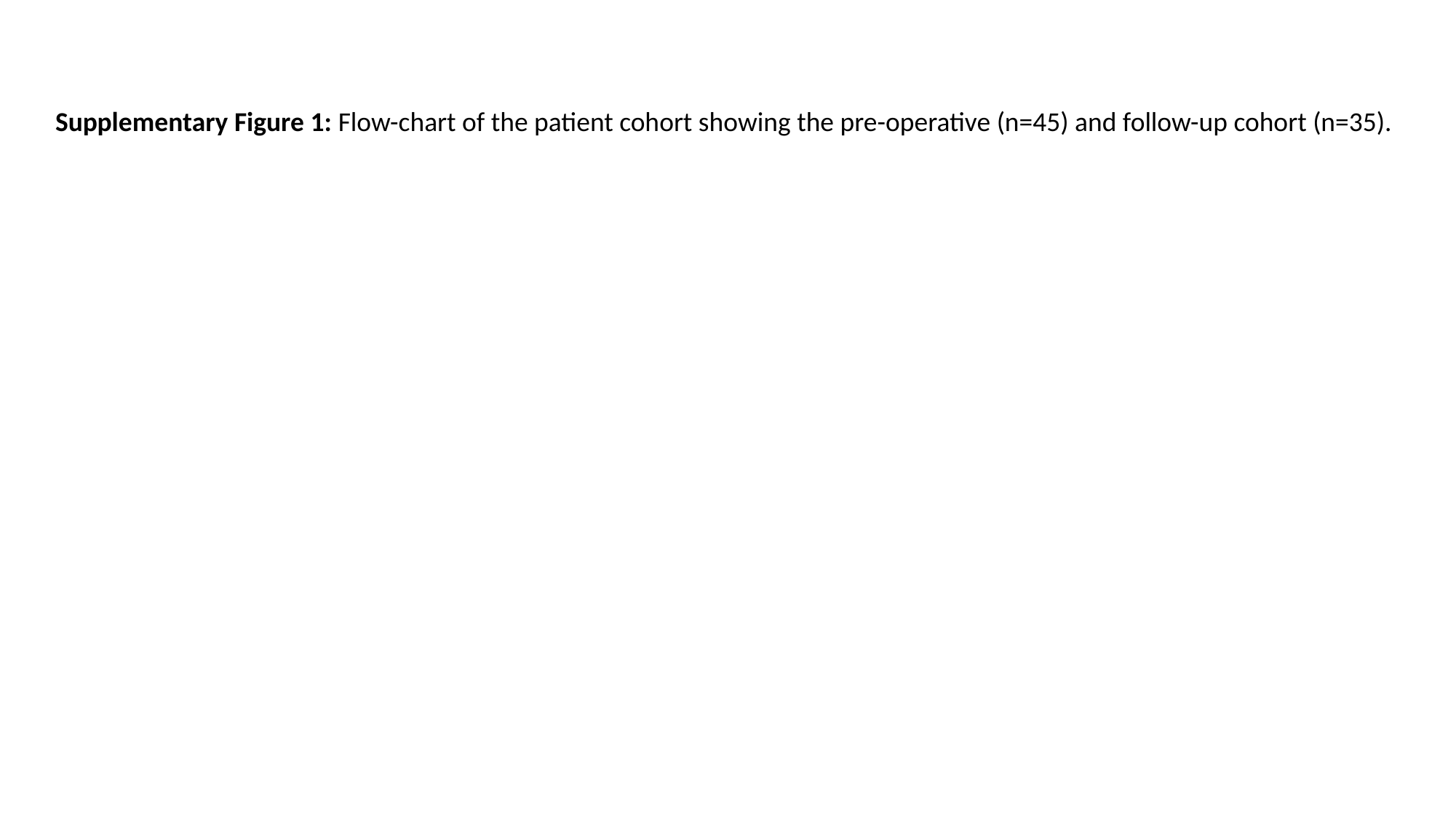

Supplementary Figure 1: Flow-chart of the patient cohort showing the pre-operative (n=45) and follow-up cohort (n=35).
